# Chronic Dislocations

**Published:** 1885-02

**Authors:** W. F. Westmoreland

**Affiliations:** Professor of Surgery in the Atlanta Medical College, Atlanta., Ga.


### Clinic Reports.

### CHRONIC DISLOCATIONS.

### SUCCESSFUL REDUCTION OF A DISLOCATION OF THE ELBOW, BOTH BONES BACKWARDS, OF TEN WEEKS STANDING.

A CLINICAL LECTURE.

BY W. F. WESTMORELAND, M. D.,

Professor of Surgery in the Atlanta Medical College, Atlanta., Ga.

Reported for The Atlanta Medical and Surgical Journal.

The next case that I present, gentlemen, is one of peculiar interest to me. The patient is a handsome young lady from the upper part of this State who was so unfortunate as to have both bones of the forearm dislocated some ten weeks since. The dislocation, as you perceive, has never been reduced. You see the olecranon process prominent on the posterior aspect of the arm, about two inches above the condyles. This state of things has existed since the injury. There was an effort made at reduction by the physician who attended her, but the reduction was not accomplished. The arm was placed on a straight splint and was kept in this position four or five weeks. At the end of this time it was found that all movement of the elbow joint was gone. You will perceive that she has no motion whatever of the joint. The arm is almost entirely useless. She says that rather than go through life with the limb in its present condition she would have it amputated.

It is needless, gentlemen, for me to tell you that this young lady has been badly treated. This injury, that could and ought to have been reduced in a few minutes at the time received, will require considerable length of time to do so now, if indeed we are so fortunate as to do so at all. These cases of old or neglected dislocations are not easily reduced. Sometimes serious consequences follow attempts at reduction. The danger lies in exerting too much force in your efforts at reduction, an important blood-vessel may be ruptured; a bone may be broken; extensive inflammation may follow; suppuration may be excited in the parts around the joint resulting in pyemia and death.

It is not unfrequently the case, gentlemen, that we find a dislocation of this character that is impossible to reduce in two or three weeks after injury. There are other dislocations, however, that are more easily reduced after a few weeks than those of the elbow. There are examples, for instance, of dislocations of the hip joint of three to six months standing having been successfully reduced.

The signs of this most common form of dislocation of the elbow are lost, in a great measure, by the treatment that has been adopted. In recent dislocations of this character you will find the limb semi-flexed. This you do not see in this case, since the arm was placed on a straight splint and kept so for four or five weeks. You do find the prominence of the condyles in front and the olecranon on the posterior aspect. The obstructions in the way of a reduction in this case are the adhesions that have been formed and the new tissue that may have been deposited in the sigmoid notch, as well as that deposited on the trochlear surface of the humerus.

The first thing to be done is to get the patient thoroughly under the influence of ether; then by extension and counter extension we hope to break up these adhesions and reduce the dislocation. This work cannot be done hurriedly, it must be done gradually, the danger to the soft parts is too great to allow of rapid stretching of the muscles. This extension, you see. is made with a compound pulley, one end attached to a rope that has been secured to the wall while the other is attached to a loop formed by a broad band that is applied around the wrist. The counter extension is made from the arm, just above the elbow. While the assistant pulls on the pulley you notice I keep my hands on the displaced bones and I find that they are gradually giving way to the great force that you notice we are exerting. Now, with all force taken off you see that the forearm can easily be carried almost to right angles with the arm. It will be impossible to reduce the deformity so long as we make extension in the direction that we are now doing. Our object now is to get the adhesions all well broken up and then we will make efforts at reduction proper. We have now brought the bones down on a line with the end of the arm bone. The bed will now be changed in order that we may get force in a different direction. You notice we attach the pulley now to a towel which we place near the joint, the hand is carried over the body. We now direct our force to lifting the coronoid process out of the olecranon fossa, and with a slight manipulation we place the bones in proper place. There is, as I have already said, new tissue formed in the sigmoid notch and on the articular surface of the bones, but this will no doubt soon be displaced and the patient will have a serviceable arm. The only dressing we apply now is adhesive plaster to keep the arm in the flexed condition you now see it in Our treatment will consist in the application of cooling lotions and the treatment of such symptoms as may show themselves.

Gentlemen, I hope I may never be called on to treat one of these neglected dislocations as the result of your treatment; if, however, I should, it would give me pleasure to testify against you in the courts in a case of malpractice. I cannot urge you too greatly to attend at once to every dislocation. Delay is dangerous.

---

NOTE.—Now, eight weeks after operation, patient has every movement of elbow joint, and has entirely recovered.—REPORTER.

